# SMARTCLOTH-Odyssey for Nutrition Education in Type 1 Diabetes: Development and Acceptability Study of a Gamified Web-Based Platform for Diabetes Nurse Educators Using a Human-Centered Design Approach

**DOI:** 10.2196/79648

**Published:** 2026-09-25

**Authors:** Guillermo Molina-Recio, Ana-Belen Ariza-Jimenez, Alberto Membrillo-del Pozo, Sandra Zafrilla-Sanchez, Jose Alvarez-Moral, Jose-Luis Velez-Leon, Rosa Gonzalez-Illanes, Rafael Molina-Luque

**Affiliations:** 1 Lifestyles, Innovation and Health Associated Group, Instituto Maimónides de Investigación Biomédica de Córdoba, Cordoba, Spain; 2 Department of Nursing, Pharmacology, and Physiotherapy,, University of Córdoba, Cordoba, Spain; 3 Pediatric Endocrinology Service, Hospital Universitario Reina Sofía, Avda Menendez Pidal s/n, Cordoba, 14004, Spain, 957010000 ext 510055; 4 Faculty of Medicine and Nursing, University of Córdoba, Cordoba, Spain; 5 Growth Study Group. Pediatric Endocrinology and Nutrition, Instituto Maimónides de Investigación Biomédica de Córdoba, Cordoba, Spain; 6 Faculty of Health Sciences, Universidad Tecnológica Atlántico Mediterráneo - UTAMED, Campanillas (Malaga), Spain

**Keywords:** diabetes mellitus, type 1 diabetes, diet therapy, diabetes education, nurse educators, human-centered design, mobile health (mHealth), gamification, mobile phone

## Abstract

**Background:**

The onset of type 1 diabetes mellitus (T1DM) in adolescence represents a challenge for patients, families, and health care professionals. Diabetes nurse educators identify a lack of adolescent-friendly educational resources, especially in nutrition education. Digital technology offers an opportunity to develop innovative tools to address these needs from a user-centered approach.

**Objective:**

We aimed to design and evaluate the acceptability of the SMARTCLOTH-Odyssey platform, an interactive web tool designed to support diabetes nurse educators in delivering nutrition education based on carbohydrate counting to adolescents with recent T1DM, evaluated from the perspective of nurse educators.

**Methods:**

A mixed study was carried out in three phases: (1) qualitative analysis through two focus groups with nurse educators in the Andalusian Community to identify barriers and needs; (2) development of the digital tool following a human-centered design methodology, incorporating narrative and gamified elements; and (3) quantitative evaluation of acceptability through an ad hoc questionnaire applied to 40 professionals who tested the platform.

**Results:**

Participants highlighted the lack of homogeneous and adapted debut materials, and the need for emotionally sensitive and visually appealing tools. SMARTCLOTH-Odyssey was rated positively in terms of usefulness, clarity, and clinical applicability. A total of 97.5% (39/40) felt it could facilitate nutrition education, and 90% (36/40) said they would use it in their practice. The platform was perceived as intuitive, innovative, and adapted to the characteristics of the adolescent patient.

**Conclusions:**

SMARTCLOTH-Odyssey constitutes a viable and well-valued proposal by nurse educators. It has the potential to improve nutrition education from the diagnosis of T1DM. Its participatory development reinforces its applicability and sustainability in real clinical contexts.

## Introduction

Type 1 diabetes mellitus (T1DM) is a chronic autoimmune disease that, despite representing only 10% of all cases of diabetes, shows a high prevalence in the pediatric and juvenile population, where its onset is especially disruptive for both the patient and their family environment [1]. In Spain, more than 30,000 children younger than 15 years of age live with this pathology, with an increasing incidence of 17 cases per 100,000 children per year and geographical disparities that place Andalusia among the communities with the highest rates [1].

At the clinical level, the metabolic effects of T1DM are well described; however, its psychosocial impact at diagnosis is less visible and more difficult to address. Families suddenly face complex responsibilities: glycemic control, insulin administration, and, most importantly, daily nutritional management using the ration diet method [2]. This process occurs in a context of high emotional burden, with a high prevalence of feelings of fear, guilt, distress, and information overload [3].

These facts are why the first moments of the debut are decisive: not only is the basis of the treatment established, but also the tone of the relationship between the patient, their family, and the health system. It is precisely here that important educational barriers emerge: difficulty in internalizing technical concepts, low nutritional literacy, poor adherence to carbohydrate counting, and a limited repertoire of tools adapted to the real capacities of families [4-6].

One of the cornerstones in managing T1DM in the pediatric population is the proper learning and application of carbohydrate-based dietary management. This approach requires calculating carbohydrate intake per meal and adjusting rapid insulin doses according to glycemic profile and physical activity [2]. While traditional teaching aids are available, many are inadequate for the stress and overwhelming emotions experienced by families during the debut [7].

Despite advances in insulin therapy and diabetes technologies, which have enabled greater flexibility in dietary management, current clinical guidelines emphasize individualized and patient-centered approaches rather than restrictive dietary patterns. However, these strategies are not universally adopted, and many patients still rely on carbohydrate counting as a fundamental tool for daily glycemic control [8,9].

Recent studies have shown that, even in advanced health systems, there are structural deficiencies in the provision of diabetes education adapted to each patient’s life and cultural stages [8]. Poor adherence to dietary recommendations is not explained by a lack of knowledge but by a disconnect between educational tools and users’ experiences [10].

This gap is exacerbated in a digitized environment: families, especially younger ones, are used to intuitive, interactive, and gamified technologies. However, many educational programs offer rigid, unidirectional, unattractive curricula [11]. In addition, diabetes nurse educators, the primary transmitters of nutritional knowledge, express the lack of specific and agreed-upon digital materials they can integrate into their care routines with children and adolescents [9].

Given this reality, human-centered design (HCD) is configured as a robust methodological alternative to solve complex health problems through technological solutions adjusted to the reality of the end user [12].

HCD is characterized by three fundamental principles: deep empathy toward the user, continuous iteration with the user’s active participation, and functional design adjusted to real contexts [13-15]. This perspective moves away from classical models of technological design, where the patient is a passive receptor. It makes him or her a coauthor of the process from the discovery phase to final validation [16,17].

Moreover, this approach has proven particularly effective in health interventions requiring behavioral changes, such as feeding or self-care in chronic diseases [18,19]. By incorporating tools such as empathy maps, rapid prototyping, cocreation with practitioners, and successive validations with real users, HCD ensures more usable, emotionally acceptable, and sustainable products in the long term [20-22].

In this context, this study aims to describe the design and evaluation process of SMARTCLOTH-Odyssey, a web-based, interactive, and gamified platform conceived to support diabetes nurse educators in nutrition education in late-onset (adolescent) T1DM.

## Methods

### Study Design

**Figure 1.**
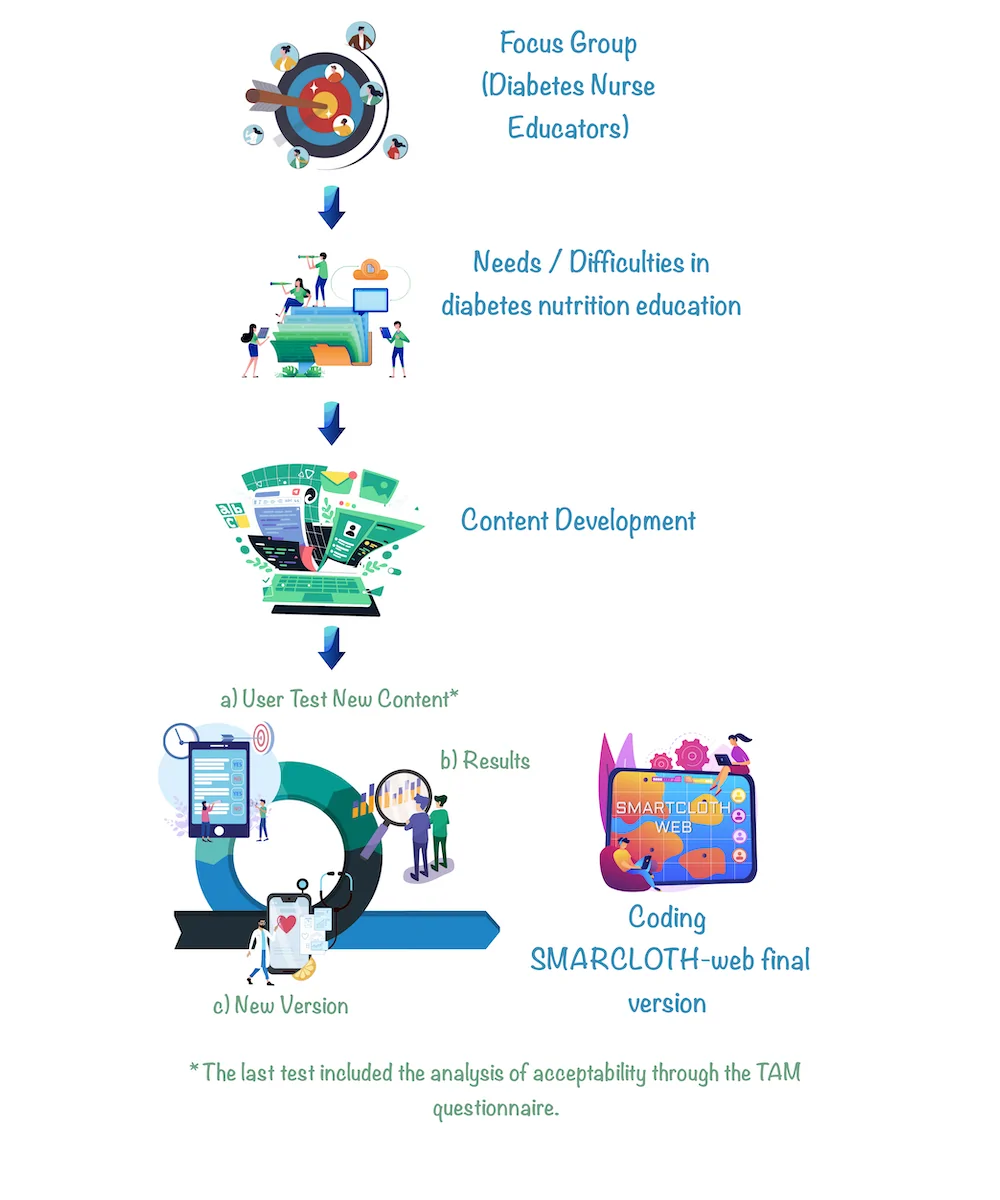
Summary of project phases. TAM: Technology Acceptance Model.

This study followed a mixed and sequential methodological approach, aligned with HCD principles and based on an iterative continuous improvement process. The development of the platform was structured in three main phases: a first qualitative phase of exploration through focus groups; a second phase of design and functional prototyping with testing; and a third phase of evaluation, where the perceived acceptance of the digital tool by diabetes nurse educators was analyzed (Figure 1). In the initial phase, the perceptions of health care professionals with direct experience in diabetes education in debut T1DM were collected. This input formed the basis for a functional design proposal, which was refined through prototype implementation and iterative review. In parallel, an evaluation was planned focusing on two key aspects: usability and technological acceptance of the platform.

### Qualitative Phase: Exploring the Needs of Diabetes Nurse Educators

The first phase of this study adopted a qualitative approach based on online focus groups to explore in depth the experiences, perceptions, and needs of diabetes nurse educators working in different provinces of Andalusia (Spain). This first approach was carried out to ensure that the subsequent development of the training program would be based on a realistic and contextualized understanding of the clinical setting in which nutrition education in the early stages of T1DM occurs.

Two focus group sessions were held on March 6 and 7, 2024, via videoconference. Focus groups were conducted by members of the research team with experience in qualitative research and diabetes education. Both sessions were attended by nurses with varying degrees of professional experience, all active in pediatric diabetes education (Table 1). The sessions were videotaped and transcribed in full, strictly respecting confidentiality’s ethical principles: personal data and interventions were anonymized and coded for subsequent qualitative analysis. Sampling was purposive and deliberately organized to ensure territorial and professional diversity. Each participant was assigned a code name, and their profile was recorded as follows.

**Table 1.** Focus group participants.

Code name	Age (years)	Experience as an educator (years)	Province
N1^a^	61	10	Cordoba
N2	56	6	Almeria
N3	40	1	Cadiz
N4	36	2	Jaen
N5	44	3	Malaga
N6	37	2 (also nutritionist)	Seville

a N: nurse.

The groups were structured around a common script, developed based on the project objectives and the conceptual framework of HCD. This script incorporated open-ended questions grouped into four broad analytical dimensions: (1) Experience using educational tools, emphasizing those incorporating technology (digital books and manuals, apps, and digital platforms). (2) Perception of more and less effective tools, both from the nurse’s point of view and the patient’s and family’s point of view. (3) Personal criteria for choosing educational resources, including technical, communicative, and emotional aspects. (4) Insight into the needs of patients and their caregivers, especially in the context of nutritional treatment debut and adherence.

Representative questions included “What kind of technological resources have you used or recommended?” “What tools do you find most effective and why?” or “What gaps have you detected in the tools available for families?” This structure allowed information on common practices and key subjective elements such as perceived barriers, associated emotions, and expectations.

The data collected were transcribed verbatim and analyzed using a thematic categorical coding strategy. This approach enabled the identification of both common and divergent patterns among participants and informed the subsequent design of the digital platform. Data collection continued until thematic saturation was reached, with no new relevant themes emerging in the final session.

The analysis followed established principles of qualitative rigor, including interresearcher triangulation and cross-review of coding. It was conducted independently by two researchers, with discrepancies resolved through discussion.

### Design and Functional Prototyping With Testing Phase

After the initial qualitative research phase, an interdisciplinary cocreation session was organized with 2 diabetes nurse educators, 1 physician specializing in pediatric endocrinology, 2 web developers, and 1 university lecturer with expertise in science education. The content blocks and the pedagogical narrative of the platform were defined in this space and structured in progressive training modules according to the findings of the focus groups.

In April 2024, the proposed structure and content were presented to the participating nurses, who validated it and offered recommendations for improvement. Three working groups were then organized to collaborate on the pedagogical and technical review of the content. Subsequently, sequential progress was made on the platform’s graphic design and programming.

Once the functional version of SMARTCLOTH-Odyssey was implemented, three structured testing sessions were conducted at different stages of the timeline: (1) October 24, 2024: diabetes nurse educators participated; (2) March 20, 2025: nursing students who had taken the nutritional care course, with knowledge of diet therapy and diabetes education participated; and (3) May 15, 2025: both nurses and pediatricians participated, to provide a more clinical and integrative view (Table 2).

**Table 2.** Participants in the testing sessions.

Session and code name	Age	Experience	Province
October 24, 2024			
N7^a^	41	6^b^	Cadiz
N8	50	15^b^	Cordoba
N9	56	6^b^	Almeria
N10	36	3^b^	Seville
N11	37	3^b^	Jaén
March 20, 2025			
NS1^c^	18	N/A^d^	Seville
NS2	18	N/A	Cordoba
NS3	19	N/A	Huelva
NS4	20	N/A	Cordoba
NS5	20	N/A	Cordoba
NS6	18	N/A	Cordoba
NS7	19	N/A	Jaén
15 May			
N11	57	36^e^	Huelva
P1^f^	48	24^e^	Cordoba
N12	59	37^e^	Huelva
N13	46	24^e^	Cordoba
N14	37	3^e^	Jaén
P2	34	8^e^	Cordoba
N15	42	17^e^	Cadiz
N16	58	14^e^	Cordoba
N17	42	21^e^	Almeria
N18	57	37^e^	Almeria
N19	43	20^e^	Almeria
N20	47	22^e^	Almeria

a N: nurse.

b Experience as an educator.

c NS: nurse student.

d N/A: not applicable.

e Experience in type 1 diabetes mellitus.

f P: pediatrics.

The March session included young students to capture a view close to that of the more digitally literate end users (teenagers). Several nurses from the initial focus groups were involved in all phases, while others participated intermittently according to availability. While most participants differed across the study phases, some nurses from the initial focus groups were also involved in subsequent testing and development activities.

The platform was developed using React (Meta Platforms, Inc), a JavaScript framework that supports responsive design, ensuring compatibility across desktops, tablets, and smartphones. Responsive behavior was verified through both simulated environments during development and subsequent testing on physical devices. User testing sessions were conducted primarily in desktop environments to facilitate screen recording and structured observational data collection using the think-aloud protocol.

**Figure 2.**
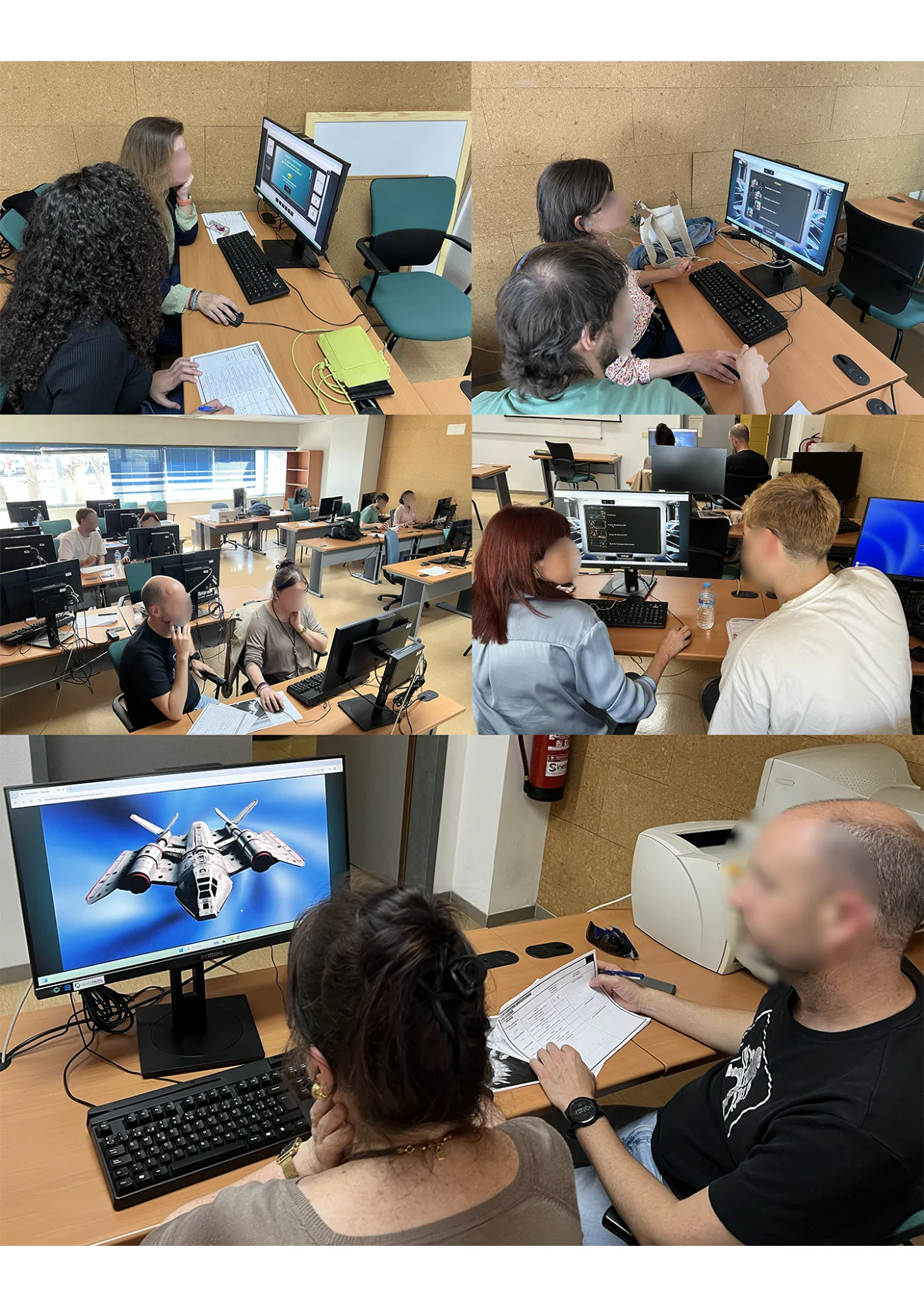
User tests on May 15, 2025.

During the sessions (Figure 2), structured usability analysis grids were used, with objective evaluation blocks and qualitative observation. Elements such as general understanding of the environment, ease of executing tasks, interaction with menus, content, and multimedia, spontaneous reactions, suggestions, and difficulties were analyzed.

The proposed tasks included creating a profile, accessing gamified content, exploring videos, answering questions, and playing some video games. In addition, the “think-aloud” technique was used, asking participants to verbalize their thoughts, perceptions, and decisions while browsing. This technique allowed us to detect areas of friction, confusion, or overload and validate elements that generated motivation or satisfaction. As previous research has shown, the “think-aloud” method is particularly useful in the functional prototyping phases of clinical applications, as it provides detailed information about usability issues from the end user’s perspective [23].

### Acceptability Study

**Figure 3.**
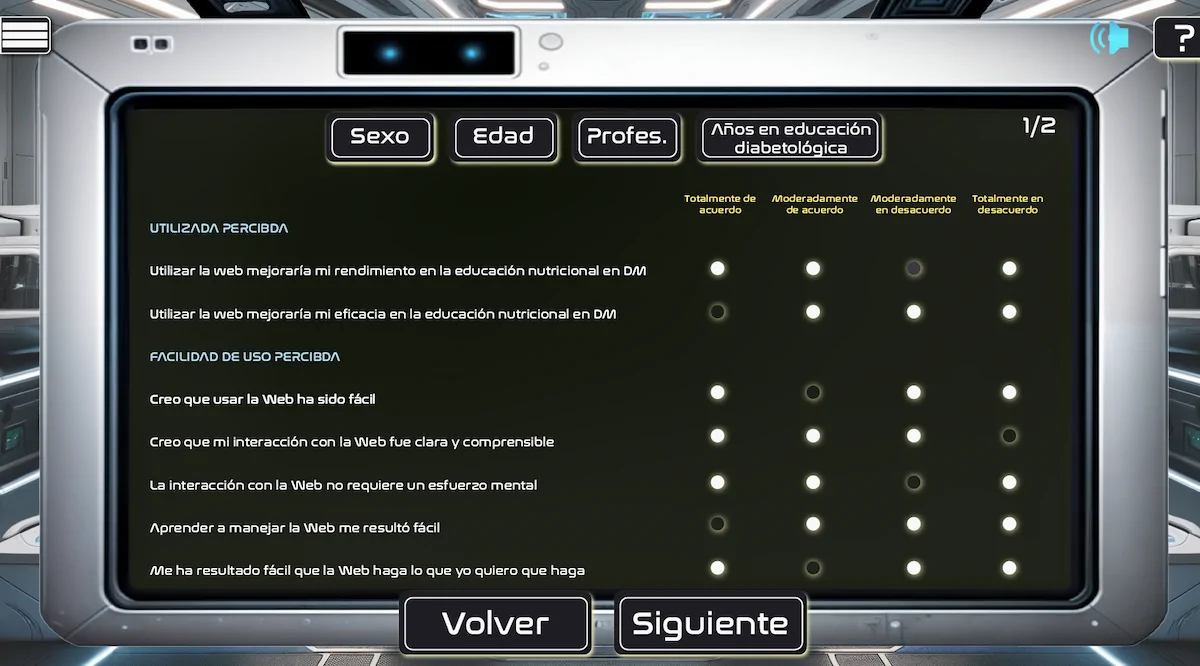
Screenshot of the website for filling in the TAM questionnaire (Spanish). Profes.: type of health care professional; TAM: Technology Acceptance Model.

At the end of the last session, participants completed the Technology Acceptance Model questionnaire adapted to the web platform, available from the side menu. This version was based on the original proposal by Davis [24] and its subsequent extension [25], adapted and validated for the project’s specific context. The questionnaire assessed the following dimensions: (1) perceived usefulness, (2) perceived ease of use, (3) attitude toward use, and (4) intention to use Multimedia Appendix 1 and Figure 3). Responses were interpreted using a Likert-type scale, with four levels of agreement with the statements in the questionnaire.

### Data Analysis

This study’s analytical approach combined qualitative and quantitative methods to ensure a comprehensive understanding of user behavior, experience, and acceptance during the different phases of the design, development, and validation process of the SMARTCLOTH-Odyssey platform. In the case of qualitative data, a thematic content analysis was conducted on the transcripts of focus group sessions and usability testing with “think-aloud.” The latter was applied during the user tests and made it possible to capture in real-time the reasoning, difficulties, doubts, or judgments that the participants expressed while interacting with the tool, generating a rich source of information on the subjective experience of use. The “think-aloud” method, widely used in user experience research, has proven particularly useful in revealing hidden elements of clinical reasoning and decision-making processes in digital health settings [26]. The audios were transcribed and anonymized, and the verbalizations were coded and classified by predefined and emergent categories related to comprehension, navigation, perceived usefulness, technological barriers, and suggestions for web improvement.

As for the quantitative data, descriptive statistics were applied to characterize the participants according to sociodemographic and professional variables (age, years of experience, etc), expressing the qualitative variables in absolute and relative frequencies and the quantitative variables through measures of central tendency and dispersion (median and IQR). To assess the technological acceptance of the platform, the responses were interpreted using a Likert-type scale with four levels, and the response percentages for each level were studied. This classification made it possible to objectively detect the platform’s level of acceptance and contrast it with the qualitative observations made in the sessions.

### Ethical Considerations

This study was conducted in compliance with the current regulations on research involving human participants in Spain, respecting the provisions established in Organic Law 3/2018, of 5 December, on Personal Data Protection and guaranteeing digital rights [27]. Likewise, the fundamental ethical principles contained in the Declaration of Helsinki [28], the Council of Europe Convention on Human Rights and Biomedicine [29], and the UNESCO (United Nations Educational, Scientific and Cultural Organization) Universal Declaration on the Human Genome and Human Rights (1997) were observed [30]. No clinical or sensitive data were collected from the participants, as the activities focused exclusively on the analysis of usability and the assessment of technological acceptance through the Technology Acceptance Model questionnaire, the completion of which was anonymized entirely through the digital platform itself. However, considering that video and audio recordings were made during the testing sessions, all participants signed an informed consent form and a specific consent form to transfer image rights.

This study is part of a broader research project developing the SMARTCLOTH ecosystem of technological solutions, which received ethical approval from the Ethics Committee for Research of the Province of Córdoba (Comité de Ética de la Investigación Provincial de Córdoba), affiliated with the Junta de Andalucía – Consejería de Salud y Consumo, at its session number 361, held on April 24, 2024. Participants received no financial compensation for their participation.

## Results

### Qualitative Phase: Exploring the Needs of Diabetes Nurse Educators

#### Overview

The two focus groups brought together a total of 6 diabetes nurse educators from different Andalusian provinces. The discussions were fluid, extensive, and richly nuanced and provided the research team with a detailed overview of actual educational practice in pediatric T1DM debut (Tables 3 and 4).

**Table 3.** Educational content distribution across SMARTCLOTH-Odyssey planets.

Planet	Content		Gamification
Newland	What happened?	General introduction to T1DM^a^, definition of glycemia and hyperglycemia, and description of the main symptoms associated with the disease.	Not included
Diabion	What is T1DM?	Explanation of the energetic role of glucose in the body, the metabolism involved, and the physiological functioning of insulin.	Questions and answers
Glucopolis	How is diabetes managed?	Discuss the importance of feeding and insulin administration as key tools for controlling blood glucose levels.	Localization of organs Labyrinths Alphabet soup
Gluconebula	How do I feel?	Analysis of the main emotional and psychological situations that people with T1DM may experience, especially in the early stages of diagnosis.	Not included
Alimentarium	What do we eat every day?	Information aimed at identifying foods according to their predominant macronutrient content (carbohydrates, proteins, and fats).	Drag and drop to sort feeding according to the main macronutrient
Dexteria Prime	Glycemic index and glycemic load	Explanation of the concepts of glycemic index and glycemic load, together with practical recommendations for their modulation in the daily diet.	Glycemic Index and glycemic load quiz
Platopia	How should I feed myself?	Guidance on appropriate food selection, frequency of consumption, and portion sizes to promote healthy habits.	Drag and drop food onto Harvard’s healthy plate.
Sportland	Playing sports with diabetes	Nutritional particularities to be considered during physical activity, adapted to blood glucose levels and other individual variables.	Not included
Racionix	How can I calculate rations?	Definition of the concept of ration and methodology for its calculation according to the type of feeding and the tools available.	Examples of dishes and foods for calculating carbohydrate servings with a calculator
Gluconix	Feeding labeling	Interpretation of nutrition labels on food products to facilitate healthy choices and correct carbohydrate counting.	Example of labels for calculating carbohydrate servings with a calculator
On the way home	Review	A series of questions related to the contents of each planet, designed to review and reinforce the learning acquired in each block.	Randomized questions from each thematic block
Earth	Menu construction and ration counting	Using SMARTCLOTH to make healthy dishes according to predefined guidelines.	Drag and drop food to build menus.

a T1DM: type 1 diabetes mellitus.

**Table 4.** Summary of the information extracted in the focus groups according to categories of analysis.

Category	Main information	Some quotes [translated from Spanish]
Variety of educational materials and strategies	Practitioners use different resources adapted to their criteria, but without a common pattern.	"And the Diabetes Foundation chart that has the weight of the raw and cooked feeding that provides a ration, too, which is also from Diabetes Foundation. That one too.” "I, for example, really like La Mesa Azul because, as I said, they are nutritionists who are involved and know a lot about diabetes and are up to date.”
Emotional barriers and adjustment difficulties	Debut is experienced with substantial emotional impact and contextual barriers that hinder learning.	“...When you are doing that (nutrition education), that the family is very overwhelmed and also that the educational approach is the part that causes the most rejection...” *“*A couple of families have told me: ‘It is very good because it gives you a lot of information, but of all the information it gives you, it is very overwhelming*.’”*
Need for homogenization and systematization	Organizing, filtering, and unifying the existing contents is essential to avoid dispersion.	"That everything is agreed upon and that we can all.” “I mean, that they are reliable websites." “...we also have a triptych that we have elaborated with a summary that we made with the consensus of all the nutrition team.”
Assessment of digital and gamified tools	Participants value the use of technologies and games as innovative educational support.	“...in other words, I understand that, in the 21st century, I am ashamed to give a printed PDF...” “...which would be that of a real solution to the problem, that is, as a useful resource for the remains, it would be a powerful app...” “...I think it would make it easier for them to put all the feeding in the app like this...”

#### Category 1: Content and Resources Used in Diabetes Education

One of the most obvious findings was the great diversity and quality of materials and approaches used by the practitioners. While all agreed on the importance of teaching the ration diet, the form and resources used were heterogeneous. Thus, N2 stated:

*If we are going to read nutrition labels and they know how to make rules of three, I don't really explain any more, but if not, I tell them to download the HC calculator application…*

*…And the Diabetes Foundation chart that has the weight of the raw and cooked feeding that provides a ration, too, which is also from the Diabetes Foundation. That one too.*

N3 and N6, on the other hand, highlighted some technology-based tools and educational websites that were well-accepted by families and nurses themselves:

*For example, I really like La Mesa Azul because, as I said, they are nutritionists who are involved and who know a lot about diabetes and are up to date.*

[N3]

*…And above all what I included was a couple of QRs with some videos that lead to the most essential, which is the video of administering glucagon, nasal glucagon, and intramuscular glucagon, so that that had to be the ABCs…*

[N6]

This finding highlights one of the cross-cutting patterns detected: information is of good quality but widely dispersed, hindering homogeneous teaching among health professionals and health centers.

#### Category 2: Barriers and Difficulties to Debut Education

All participants pointed to the emotional challenge of educating families and patients at the very moment of diagnosis, a context of high emotional burden and vulnerability. Similarly, providing a large amount of information at once, even entire basic books to be read quickly, is seen as another source of stress. N6 shared:

*And I find it tedious and very tiresome…*

*…They come in a bit overwhelmed. "I have not managed to read the book". We reply, "Well, don't worry…”*

In the same vein, N5 argued that:

*…When you are doing this (nutrition education), the family is overwhelmed, and also, the educational approach is the part that causes the most rejection…*

*I mean, it’s a difficult part, at the beginning and afterwards. It’s like a part that causes a lot of rejection, the counting of rations.*

*The educational approach is not done very well either, in my view or in my opinion. And it generates a lot of confusion and insecurity for the family and the patients.*

In the same vein, N3 indicated that:

*…a couple of families have told me: “it’s very good because it gives you a lot of information, but of all the information it gives you, it’s very overwhelming.”*

In addition, some participants expressed reluctance to use materials for a variety of reasons, in some cases due to lack of knowledge, in others due to lack of updates, or insecurity about the data they provide.

*Because it’s true that the material we have I think is a little bit obsolete, which is what N5 said. It’s from 2018.*

[N6]

*I have been told about some apps that use the camera on your mobile phone to tell you how many portions the food has, the dish in front of you, but what I have been told, and I can't check it, because I haven't done it, is that they are not reliable, so they are not recommended.*

[N2]

*I think that the issue of new technologies… many times we try to keep up to date with all the technologies that are coming out and a lot of things emerge and I think that there, at least I do falter a little… they, as the colleagues say, are very attentive to everything that comes out… All that information I can't… I personally don't have the slightest idea of everything that is emerging…*

[N3]

#### Category 3: Need for Homogenization and Shared Tools

One of the clearest consensuses was the need for a tool that serves as an everyday, accessible, shared, and reliable basis, as stated by N3:

*I think that in the end we need to reach a consensus and that all the materials we use should be… what our colleague mentioned earlier. That everything is agreed upon and that we can all…*

*I mean, that they are reliable websites, that it doesn't happen like you have to pay this thing, "you have to pay I don't know how much to enter…”*

This need for homogenization without rigidity was constant in both groups, highlighting an opportunity to improve equity in educational care after the debut of T1DM and not forcing educators, patients, and their families to use a wide variety of resources scattered on the web or printed material. Even in some nutrition units, they had tried to reach a consensus and condense basic information:

*And then, before discharge, we also have a triptych that we have prepared ourselves with a summary that was agreed upon by the entire nutrition team and all the doctors, a joint effort.*

[N6]

#### Category 4: Valuing the Use of Digital and Gamified Tools

The perception of digital tools and their potential was favorable, especially concerning adolescents. A description of the perception of the work they are sometimes forced to use during the consultation stands out:

*That it doesn't exist. I mean, that there isn't, because, for example, if I have material that I know is useful, for me, the part… whether it’s publicity or not, I don't…. I mean, I understand that, in the 21st century, what I'm ashamed of is giving out a printed PDF. I mean, I promise you… When from your mobile phone, you can see from China how your garden is, I think the solutions we give are prehistoric.*

[N6]

*…and then, for me, the next part, which would be to really solve the problem, that is, as a useful resource for the leftovers, would be a powerful, well-formed app in terms of ration counting…*

[N5]

*I think it would make it easier for them to enter all the feedings in the app and they could also visually see how many portions they have… I would love, at least, the little time we spend with the patients, for it to be efficient and effective…*

[N3]

#### Conclusions of the Qualitative Analysis

Ultimately, several key points emerged from the analysis that were shared by the group and guided the next design phase: (1) there is a wealth of valuable and reliable information and resources; (2) this information is widely dispersed and not always organized or structured in a way that is useful for reuse; (3) each nurse uses the materials and methods that are most comfortable and effective for them, leading to inequalities in teaching; and (4) there is a perceived need for a standard digital tool, which brings together key content in a didactic, straightforward, and adaptable way.

#### Design and Functional Prototyping With Testing Phase

##### Design and Prototyping

Once all the information obtained in the focus groups had been collected and analyzed, we proceeded to define the visual section of the website, the thematic blocks that would make up the educational proposal, the way in which the information would be presented, and the materials necessary to offer support and facilitate the resolution of the patients’ doubts.

In this sense, it was agreed that the visual design of the educational proposal would follow a spatial theme. From the perspective of the research team, and in line with the opinions expressed by the experts participating in the process, it was considered that a visual approach that avoided excessively childish elements was more appropriate for the clinical setting in which nurse educators would use the tool during diabetes education consultations with adolescent patients. As the platform is intended to be used by nurses as an interactive educational resource in direct consultation, the visual design needed to be professional and credible enough for the health care professional, while remaining visually engaging and age-appropriate to capture and maintain the attention of adolescent patients throughout the educational session.

Once the visual aspect was agreed on, the contents to be included in the educational proposal were selected, as well as the way they should be grouped, the way they should be presented, the elements of gamification to be incorporated, and the complementary material that could be developed to support the patients’ educational process. In order to give coherence to the proposal and prevent the website from being a simple compilation of information, the research team opted to structure all the contents through a narrative in which the patient himself would play a leading role. This narrative consists of an intergalactic journey in which the protagonist must gather information and assemble the pieces of his ship in order to return home safely. In addition, during the development process, special emphasis was placed on creating names, visual elements, and environmental features with a creative approach, aiming to foster a playful and emotional connection with the users to achieve greater acceptability of the tool. At this stage of development, the educational proposal was named SMARTCLOTH-Odyssey.

Within the narrative framework of the intergalactic journey, the educational content was structured around a series of planets, each of which addresses different topics related to the diagnosis of diabetes, its impact on patients, and the nutritional approach to treating the disease. It was also determined that the content would be presented through short videos, in which different characters would explain the information corresponding to each block, using both voice-over and subtitles to improve accessibility. In addition to the audiovisual material, a game was incorporated at the end of some exercises, aiming to review and consolidate the knowledge acquired. Table 3 presents the name of each planet and a summary of the content covered in each.

It should be noted that access to each planet is only unlocked after passing the content of the previous block. This requirement allows the user to review the knowledge acquired before advancing along the training itinerary. This progressive mechanic reinforces sequential and structured learning within the playful environment.

To reinforce the learning process, supplementary materials were proposed in addition to the main content. All the content related to the planets and other additional topics, such as the use of sweeteners, was compiled in PDF format. Most of these documents were also visually adapted to the space theme, thus maintaining the aesthetic coherence of the educational proposal.

As explained above, an active participation methodology was adopted in this phase of the project based on cocreation with nursing professionals specialized in diabetes. Once the fundamental structural aspects of the website had been defined, a series of sketches was drawn up that included the essential ideas of the proposal in terms of visual design, minimum content, and the way in which the information would be presented. Subsequently, in April 2024, a validation session was held with the same nurses who had previously participated in the focus group. During this session, the professionals provided feedback on the proposal, mostly validating the presented elements and positively commenting on the ideas. Concerning the conceptual proposal, the only noteworthy observation made by the group of professionals was the recommendation to replace the expression “danger, nutrition labeling!” with “attention, nutrition labeling!” due to the negative connotation conveyed by the first formulation.

Once the conceptual basis for SMARTCLOTH-Odyssey had been established, working groups comprised the researchers and nurses who had participated in the focus groups. Each group was assigned the development of the theoretical and recreational content corresponding to one or more planets, depending on the area of expertise of each nurse and researcher. This participatory approach sought to avoid unidirectionality in decision-making, given that ideas proposed exclusively by a single person can limit creativity. This work was planned to be developed over eight sprints (Table 5), which also included key elements of web programming.

**Table 5.** Development task distribution across SMARTCLOTH-Odyssey sprints.

Sprint	Development tasks
1	Databases Backend Web skeleton User account screens.
2	What happened? What is diabetes mellitus? How is diabetes mellitus managed?
3	How do I feel?
4	What do we eat every day? Glycemic index and glycemic load
5	How should I feed myself every day? Playing sports with diabetes
6	How can I calculate rations? Feeding labeling
7	Review Menu construction and ration counting
8	Final details and review

Each working group reviewed the existing materials related to therapeutic education in diabetes, corresponding to the thematic blocks assigned to them. The aim was to define the final contents and agree on the final design of each planet. This review sought to ensure both the clinical and educational relevance of the material, actively integrating the practical experience of the nursing professionals in the construction of the didactic narrative. Once this phase was completed, each group developed the educational materials, which were then transformed into explanatory videos by a graphic designer. These videos feature characters generated by artificial intelligence, with synthesized voices, and are accompanied by illustrative images designed manually by the designer herself.

In addition to all of the above, and as mentioned at the beginning of this section, aspects related to the web platform’s specific programming and the information layout’s design were also addressed. In this sense, work was progressively carried out on developing key interface elements, such as the side menu of options, which offers direct access to the different functionalities as they are unlocked. Contextual aids were also implemented on the different screens to ensure intuitive navigation and prevent patients from becoming disoriented while using the tool.

The sequential development of SMARTCLOTH-Odyssey allowed it to be tested three times at different stages of the design and implementation process. The main results obtained in each evaluation are presented below.

##### Test 1

**Figure 4.**
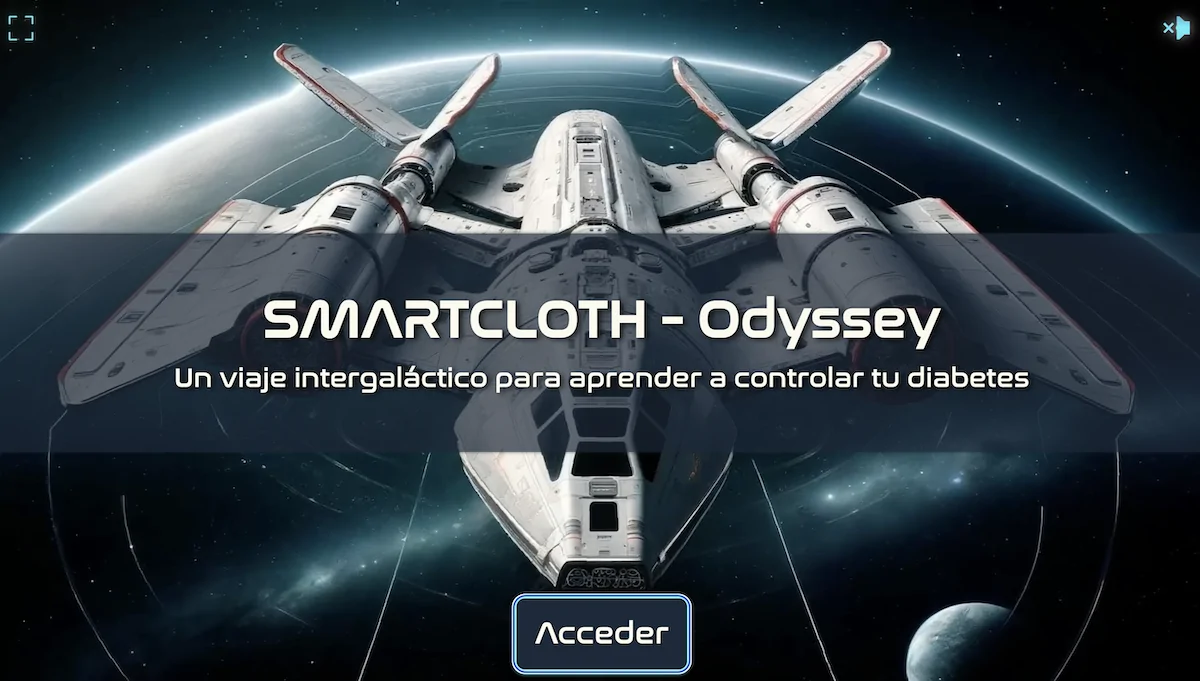
SMARTCLOTH-Odyssey home screen.

The first test was conducted in October 2024, using an early version of SMARTCLOTH-Odyssey (Figure 4). Despite being an initial version, this allowed us to gather the participating professionals’ first impressions and identify the most relevant malfunctions. To this end, 17 specific tasks were designed, focusing on specific aspects of navigation and interaction with the elements available on the platform up to that point. During this phase, the professionals had the opportunity to explore and evaluate the (incomplete) content corresponding to the Newland and Diabion planets.

**Figure 5.**
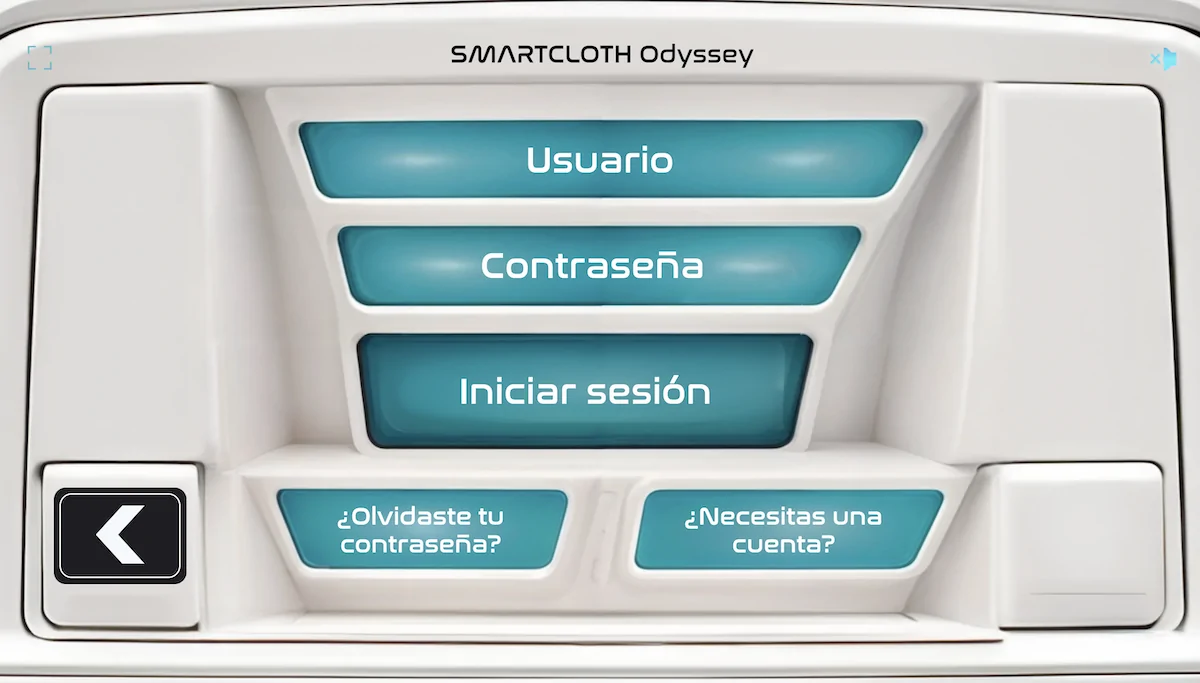
SMARTCLOTH-Odyssey login (Spanish).

In general, the problems identified during this first test were similar among all participating nurses. Most of them tried to log in to the platform without having previously registered, which raised doubts about whether it was necessary to take any previous steps to access the content (Figure 5). Difficulties were also observed in locating the game’s sound control icon, and on some occasions, navigation problems were encountered, as participants were not completely clear on which elements they should click on to progress in the interaction with the website.

However, apart from the aforementioned problems, the nurses did not report significant difficulties navigating the various interactive elements available. They could handle functions such as changing the volume of the videos, full-screen display, reducing the size of the videos, advancing the playback, and interacting with the first game developed (based on questions and answers about T1DM) without any problems.

Given that this was an elementary version, no major problems were identified in terms of content or programming, as no highly complex elements had yet been incorporated. However, one aspect unanimously pointed out by the professionals was the excessive volume of the music and the perception that some of the explanatory videos were too long.

Finally, despite being a preliminary version of the platform, the participants expressed their agreement with the contents included and the way in which they were presented. They also began to visualize the tool’s potential as a complementary resource to reinforce therapeutic education in diabetes, both during the patient’s hospitalization and in the postdebut period, once at home.

##### Test 2

The second test was conducted in March 2025, using a significantly more advanced SMARTCLOTH-Odyssey, including the planets Newland, Diabion, Glucopolis, Gluconebula, and Alimentarium. Adjustments were also implemented in response to observations made during the first test. In particular, the length of the new videos was shortened as much as possible while maintaining the rigor of the explanation of the contents, and the default volume of the music was modulated to avoid discomfort during navigation. This evaluation was carried out with nursing students who, having completed coursework in nutritional care and diabetes education, brought a dual perspective: as future health care professionals with specific knowledge of T1DM educational processes, and as digitally literate young adults whose familiarity with interactive technologies provided valuable insights into the platform’s usability and navigability. The test comprised 54 specific tasks focused on specific aspects of navigation and interaction with the available elements, through which the participants not only interacted with the educational contents of the planets, but also could also explore additional functionalities, such as the website’s side navigation menu.

In this second test, given that more complex functionalities had already been incorporated, especially in relation to some of the new games, a greater number of incidents were observed during the task development. The main problems detected in this evaluation phase are described below.

One of the most relevant aspects was the persistence of difficulties related to login. Participants were still unsure whether or not to create an account to access the platform, which led to some confusion at the beginning of the browsing experience.

*Do I create an account? Do I really believe it?*

[NS2]

*Do I need to create an account?*

[NS4]

Concerning the observations made during the first test, it was again pointed out that the base volume of the music should be considerably lower. In some cases, it was reported that the music overlapped or interfered with the characters’ voices, making it difficult to understand the content. Additionally, situations were detected, for example, during the playing of games, where it was impossible to modulate the volume, which negatively impacted the user experience. Several participants explicitly verbalized this limitation during the test sessions.

*Where is the icon of music?*

[NS3]

The suggestion to shorten the length of some videos was also reiterated, as several were too long and too heavy to hold users’ attention. Among the suggestions for improvement, the possibility of slightly increasing the playback speed of the characters’ voices was also mentioned. As it is a synthesized voice, its pace was perceived as too slow, which hindered the fluidity of the content’s presentation.

*The characters, perhaps they speak too slowly.*

[NS6]

Despite the above-mentioned observations, there was a broad consensus among the participants that the design, music, and voice explanations contributed significantly to making the content easy to understand. The visual component, in particular, was highly valued by the students, who highlighted its aesthetic appeal and its ability to facilitate understanding of the concepts addressed.

*It’s really cool, I love it.*

[NS1]

*This has got a job to do, my goodness!*

[NS5]

**Figure 6.**
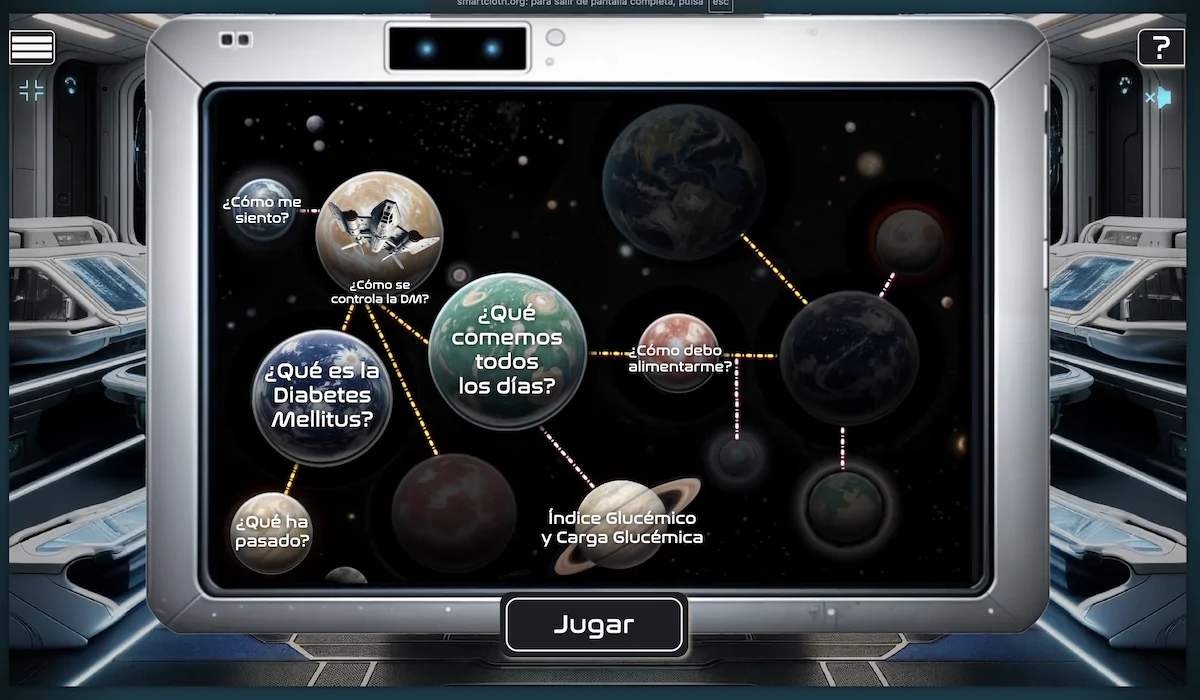
SMARTCLOTH-Odyssey star map.

Notwithstanding the positive aspects mentioned above, certain elements were identified that hindered the interaction between the students and the game environment. At some points, videos were blocked or interrupted, which prevented them from continuing normally and made it necessary to restart the navigation. Additionally, in certain playlists, it was unclear which videos had already been viewed and which had not, leading to confusion about the next step to take and slowing down progress in the game. Another notable problem was the lack of planet identification on the star map. When the in-game characters indicated that a specific planet was to be reached, the students were not clear on where to click, which also hindered the user experience (Figure 6).

This was also verbalized during the tests:

*I don't know where Glucopolis is.*

[NS5].

About the above, it was proposed that each planet incorporate its name in visible form and a brief description of the content the user can expect to find in it to facilitate navigation and orientation within the platform.

In terms of games, this component was by far the one that generated the highest number of incidents during testing. The problems most frequently mentioned by participants included the following: (1) monotony in some games, resulting from the need to repeat the same actions several times; (2) excessively strict or precise requirements to achieve the stated objectives; (3) limited time for resolution, especially in those tasks that required further reflection or consultation of supplementary material; (4) difficulty in accessing the correct answers after completing a quiz-type questionnaire; (5) technical errors or bugs that blocked the game and prevented its completion; (6) lack of clear indicators on the time remaining to complete the activities; and (7) lack of clarity in the instructions as to which input devices (mouse, keyboard, or both) could be used in which game.

The participants explicitly verbalized many of these problems, leading to frustration during the user experience.

*I didn't know I was counting the time.*

[NS3]

*Why do I have to put the organs in three times?*

[NS5]

*I struggled to identify what I needed to move.*

[NS7]

Despite this, it was stated that the games were well-liked:

*I liked the game.*

[NS7]

*The mini-games are very cool.*

[NS6]

**Figure 7.**
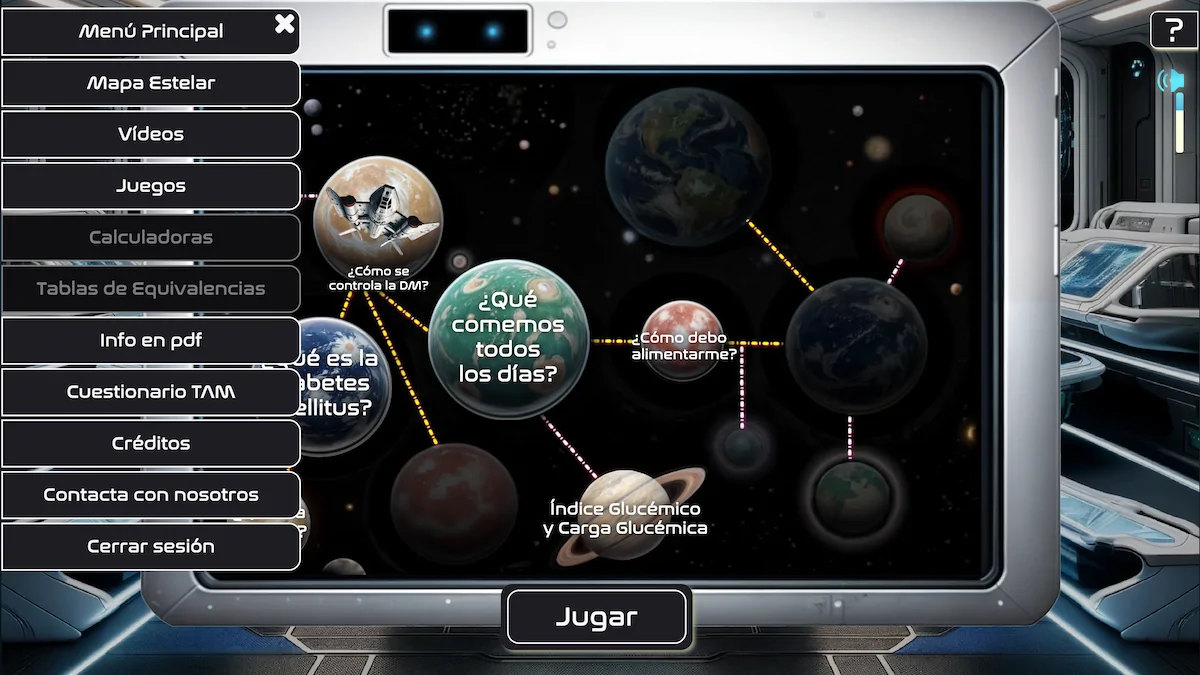
SMARTCLOTH-Odyssey side menu. TAM: Technology Acceptance Model.

Finally, another element valued by the participants was the access to the side menu (Figure 7). This menu not only allows direct access to all the videos and games that are unlocked throughout the adventure but also includes PDF files that contain, in textual form, all the information explained in the audiovisual content. This functionality was particularly appreciated for its usefulness as a resource for consultation and reinforcement of learning.

These PDFs generated a very positive response from the students, as they were very appealing to them because of the visual aspect, adapted to the aesthetics of the website:

*It makes me want to pass it on to my friend with diabetes.*

[NS2]

In general, and once again, despite the technical problems detected, SMARTCLOTH-Odyssey received numerous positive evaluations from the participants. This is evidence of a favorable evolution in the tool’s development, and the work has been consolidated since the first test.

##### Test 3

The third and last test was carried out in May 2025, using the version of SMARTCLOTH-Odyssey that, after this final evaluation, would be considered the definitive version of this project. In addition to the planets and elements already described in the second test, this test version incorporated the new planets called Dexteria Prime and Platopia. However, it was not possible to include all the content modifications suggested in the previous test, as the development period between the two phases focused on the introduction of the new materials and the resolution of technical problems that affected the correct functioning of the platform, such as errors in the playback of videos or failures in the execution of some games. This final test involved the participation of professionals directly involved in therapeutic education processes and was structured around a total of 17 tasks to evaluate general aspects of the website’s functioning.

In this last test, the professionals made particularly valuable contributions, mainly from a clinical perspective. Their experience allowed them to enrich the way some of the information is presented, making it more understandable and more relevant for the end user, and detecting minor errors that contributed to refining the contents and improving the overall quality of the tool.

Participants generally expressed a positive level of satisfaction in terms of visual aspects. However, some confusion in navigation was identified, especially between the main menu and the star map. In particular, it was noted that users tended to confuse planets already visited with those not yet explored because they all had legible labels. It was therefore suggested that the names of nonaccessible planets be presented in a more blurred or less contrasting way to make it easier to differentiate between them. In addition, some participants pointed out that specific special effects, although aesthetically appealing, could distract patients while using the platform, potentially affecting their concentration or understanding of the content.

In terms of content, only one specific recommendation was made to incorporate relaxation techniques or guidelines for emotional regulation to complement the educational approach of the tool. Regarding the games, the participants pointed out that, in the question-and-answer activities, some of the options were too long, which could hinder active participation and understanding by the patients. It was also suggested that, in case of failure, only the failed questions should be repeated rather than repeating the whole set, as the latter could lead to a feeling of monotony. It was also suggested to improve the immediate feedback during the game so that correct answers are shown in green and incorrect answers in red, thus facilitating visual learning. Finally, an error was detected in one of the questions on fasting blood glucose levels, where the value indicated was 70‐150 mg/dl, when it should be 70‐180 mg/dl.

Concerning supplementary material, the professionals highlighted the importance of improving the visibility of key resources, such as glycemic index tables, glycemic load, and food equivalencies. They considered it essential that these documents be placed in a more accessible location, given their practical value for dietary management in patients with diabetes. Regarding PDF files, some participants suggested incorporating pagination to facilitate printing and the availability of a black-and-white version to promote more environmentally and economically sustainable practices. Finally, it was recommended that the visual accessibility of the side menu be improved by adding illustrative images next to the text of each option. This measure could facilitate understanding the content and navigation within the platform, especially for users with lower digital literacy or visual difficulties.

Finally, some technical problems of particular relevance were identified. In particular, it was observed that account recovery e-mails did not reach users correctly, making it difficult to restore access in cases of forgotten credentials. Additionally, on the login or account creation screen, error messages, for example, “credentials are not correct,” were displayed without a contrasting background, which caused them to overlap with the interface background and make them difficult to read. Such technical issues can considerably negatively impact the user experience, as they hinder access to the platform and could reduce patient adherence to continued use of the tool. Although this is not a problem as such, it was also recommended that, to improve accessibility to information after a first viewing, when reaccessing an already visited planet, the user should be able to directly select the content they wish to consult, without the need to replay the whole block from the beginning.

In summary, this last test allowed for identifying and correcting several problems that had not been detected in previous phases and adjusting minor errors in the contents. However, it is particularly relevant to highlight that, throughout the different tests, no substantial criticisms were registered about the pedagogical content of the proposal. On the contrary, most participating professionals expressed their surprise at the tool, highlighting its potential as a complementary resource in daily clinical practice and therapeutic education processes in T1DM.

*The tool and the PDFs apply to daily clinical practice.*

[N14]

*Access to scientifically backed information is critical.*

The final result after the design, prototyping, and testing phase can be seen on the website [31]. However, as will be explained in the limitations, although the theoretical content is fully developed, the web programming of the planets was interrupted in Platopía due to time and funding problems.

### Acceptability Study

Finally, 23 health care professionals participated in the questionnaire. The median age was 44 (IQR 38‐53) years, and the median experience in diabetes education was 12 (IQR 7‐20) years. In terms of professional distribution, the majority of participants were diabetes nurse educators (n=19), followed by pediatricians (n=3) and 1 student nurse.

### Perceived Usefulness

The responses showed a high degree of agreement regarding the platform’s usefulness. A total of 73% (19/26) of the participants strongly agreed that using the website would improve their performance and effectiveness in nutrition education in T1DM, while 27% (7/26) moderately agreed.

### Perceived Ease of Use

This dimension assessed the users’ experience of interacting with the platform. The scores showed that 57% (15/26) of the responses indicated a level of total agreement with statements such as “using the website was easy” or “I found it easy for the website to do what I want it to do.” The remaining 43% (11/26) were “moderately agree.” These results indicate an overall good perception of the tool’s usability, but also suggest areas for potential improvement in the user experience to reach the highest levels of usability and user experience.

### Attitude Toward Use

The questionnaire reflected a very positive attitude toward the use of the digital tool. A total of 96% (25/26) of the responses were grouped in the “strongly agree” category, compared to 4% (1/26) who stated, “moderately agree.” This result shows an evident willingness on the part of the participants to integrate this type of tool into their regular clinical practice.

### Intention to Use

Regarding the intention of future use, 88% (23/26) of the participants indicated that they totally agreed with its continued use if they had stable access to the platform. In comparison, 12% (3/26) of the participants were moderately in agreement. This finding is consistent with the results observed on attitude and reinforces the feasibility of implementing the digital resource developed.

## Discussion

### Principal Findings

This study aimed to design and assess the acceptability of SMARTCLOTH-Odyssey [31], a web tool with interactive elements and gamification designed to support diabetes nurse educators in delivering therapeutic nutrition education on T1DM during the debut stage in adolescents. Specifically, the educational proposal focuses on fundamental aspects related to the diagnosis, the characteristic symptoms, the emotional impact derived from the changes brought about by the disease, and, especially, the dietary approach and food management as the central axis of the intervention.

The results of this study show that diabetes nurse educators identify multiple limitations in addressing therapeutic nutrition education in adolescents with T1DM. They point to the lack of visual, interactive, and adolescent-friendly materials. In addition, they highlight the scarcity of resources specifically designed for the time of debut, which in many cases forces them to reuse materials designed for other population profiles.

These barriers are consistent with what has been described in other studies. In this regard, it has been documented that adolescents with T1DM prefer digital tools that are accessible, dynamic, and adapted to their everyday technological environments, such as mobile apps or gamified environments, rather than traditional materials focused on the adult or clinical setting [27-30]. Furthermore, several studies highlight that health care professionals, including diabetes educators, perceive the lack of validated digital content in real clinical settings as a limitation to integrating these tools into their daily practice [32]. In this regard, initiatives such as the development of emotional support chatbots [28], gamified platforms [30], or educational programs based on mobile messaging [33] have been well accepted by both patients and professionals.

However, in many cases, these digital resources have been designed without the structured involvement of diabetes educators, which may limit their real applicability in the clinical setting [34,35]. This paper addresses this gap by developing a tool cocreated with professionals directly involved in therapeutic education, with the aim of providing nurse educators with a structured, reliable, and clinically applicable resource to support adolescent patients from the moment of diagnosis. This approach is in line with current recommendations that digital educational programs should be human-centered and adapted to the real clinical context [32,34,35].

In the case of this work, HCD was used; that is, the professionals participate in all phases of development as key users, intending to ensure not only that it is adapted to the clinical setting but also that the tool is practical, realistic, and adaptable to the different situations that arise in their usual clinical practice. This type of design has demonstrated multiple benefits in digital health, especially for diabetes. When end users are integrated early in the development process, acceptability, usability, and adherence to educational interventions significantly increase [36,37]. In the case of adolescents, this approach has shown concrete improvements in treatment understanding, motivation toward self-care, and family engagement [38,39].

In addition, participatory design allows the language, interface, and content to be tailored to the end user’s level of health literacy, reducing barriers to access and personalizing learning [40]. It has also been observed that this approach facilitates the integration of tools into clinical workflows by responding to the fundamental dynamics of the care environment [37,41]. In the case of this platform, the fact that it has been designed jointly with professionals who lead education in the debut of T1DM anticipates a greater appropriation of the resource, which could translate into sustained use over time and an improvement in the educational experience of adolescents and families [42-46].

### Limitations

Due to time and funding constraints, this study’s main limitation is that SMARTCLOTH-Odyssey has not yet been developed in its final version. Although practitioners have conceptually validated the prototype, it has neither been implemented nor evaluated in a real clinical setting, nor has it been directly tested with adolescents with T1DM at the time of debut.

It should be noted that SMARTCLOTH-Odyssey has been conceived primarily as a tool for use by diabetes nurse educators within their clinical consultations, providing a unified, structured resource to support therapeutic nutrition education at the T1DM debut. In this study, nurse educators were therefore considered the primary end users, and the HCD methodology was applied accordingly, with their active involvement across all phases of development and evaluation.

Nevertheless, the platform also has potential for direct use by adolescent patients with T1DM, who may access the same educational content (videos, gamified modules, and PDF summaries) independently after the clinical encounter. Evaluating usability and technological acceptance from the perspective of this secondary user group constitutes an important future line of work, which will require specific adaptations to the platform, including a revised acceptability questionnaire oriented toward patients rather than health care professionals. This second phase of evaluation will be necessary to fully characterize the platform’s educational impact and ensure its alignment with the needs of adolescent users.

This second phase of evaluation, involving adolescents with T1DM as active participants, will be essential to fully validate the platform’s educational impact and ensure its alignment with the real needs and preferences of this population. Furthermore, incorporating adolescents as co-designers in this future phase would allow the HCD process to be extended to this secondary user group, complementing the HCD work already carried out with nurse educators. It should be emphasized, however, that the primary mode of use of SMARTCLOTH-Odyssey is nurse-mediated: the platform has been designed to be used by diabetes nurse educators within their clinical consultations as a structured educational resource, and it is in this context that the HCD methodology has been rigorously and systematically applied throughout this study.

Finally, the sample, focused on a single autonomous community, limits the generalizability of the results to other health care contexts. Although nurse educators have been considered key users at this stage, including the patient’s perspective in future validation stages will be necessary to adapt some design elements to their real needs and ensure its educational effectiveness.

### Conclusions

SMARTCLOTH-Odyssey [31] responds to the need for a structured digital tool that enables diabetes nurse educators to deliver effective and engaging nutrition education to adolescents debuting with T1DM, aligned with real clinical practice. Based on user-centered design, its development has allowed the integration of nurse educators’ perspectives, resulting in high acceptability and perceived feasibility. The platform shows potential to improve nutrition education from diagnosis and facilitate the work of health professionals through an attractive, understandable, and clinically useful resource.

## Supplementary material

10.2196/79648 Multimedia Appendix 1 Adaptation of the technology acceptance model for the SMARTCLOTH-Odyssey web acceptability study.

## Abbreviations

HCD: human-centered design
T1DM: type 1 diabetes mellitus
UNESCO: United Nations Educational, Scientific and Cultural Organization

## References

[1] Magliano DJ, Boyko EJ (2025). IDF Diabetes Atlas.

[2] Gillespie SJ, Kulkarni KD, Daly AE (1998). Using carbohydrate counting in diabetes clinical practice. J Am Diet Assoc.

[3] Lansing AH, Berg CA, Butner J, Wiebe DJ (2016). Self-control, daily negative affect, and blood glucose control in adolescents with type 1 diabetes. Health Psychol.

[4] Bayram S, Kızıltan G, Akın O (2020). Effect of adherence to carbohydrate counting on metabolic control in children and adolescents with type 1 diabetes mellitus. Ann Pediatr Endocrinol Metab.

[5] Tascini G, Berioli MG, Cerquiglini L (2018). Carbohydrate counting in children and adolescents with type 1 diabetes. Nutrients.

[6] Uliana GC, Carvalhal MMDL, Berino TN (2022). Adherence to carbohydrate counting improved diet quality of adults with type 1 diabetes mellitus during social distancing due to COVID-19. Int J Environ Res Public Health.

[7] Gómez-Peralta F, Menéndez E, Conde S, Conget I, Novials A (2021). Clinical characteristics and management of type 1 diabetes in Spain. The SED1 study. Endocrinol, Diabetes Nutr (English ed).

[8] Annan SF, Higgins LA, Jelleryd E (2022). ISPAD Clinical Practice Consensus Guidelines 2022: nutritional management in children and adolescents with diabetes. Pediatr Diabetes.

[9] ElSayed NA, Aleppo G, Aroda VR (2023). 5. facilitating positive health behaviors and well-being to improve health outcomes: standards of care in diabetes-2023. Diabetes Care.

[10] Carratalá-Munuera MC, Gil-Guillen VF, Orozco-Beltran D (2013). Barriers associated with poor control in Spanish diabetic patients. A consensus study. Int J Clin Pract.

[11] Molina-Recio G, Molina-Luque R, Romero-Saldaña M (2021). The importance of knowing and listening to all those involved in the design and use of nutrition mobile apps. Getting to know the Great GApp. Nutr Hosp.

[12] Altman M, Huang TTK, Breland JY (2018). Design thinking in health care. Prev Chronic Dis.

[13] Dopp AR, Parisi KE, Munson SA, Lyon AR (2020). Aligning implementation and user-centered design strategies to enhance the impact of health services: results from a concept mapping study. Implementations Sci Commun.

[14] Chen E, Neta G, Roberts MC (2021). Complementary approaches to problem solving in healthcare and public health: implementation science and human-centered design. Transl Behav Med.

[15] Crowe B, Gaulton JS, Minor N (2022). To improve quality, leverage design. BMJ Qual Saf.

[16] Richardson S, Lawrence K, Schoenthaler AM, Mann D (2022). A framework for digital health equity. NPJ Digit Med.

[17] Roberts JP, Fisher TR, Trowbridge MJ, Bent C (2016). A design thinking framework for healthcare management and innovation. Healthc (Amst).

[18] Sieck CJ, Sheon A, Ancker JS, Castek J, Callahan B, Siefer A (2021). Digital inclusion as a social determinant of health. NPJ Digit Med.

[19] Levander XA, VanDerSchaaf H, Barragán VG (2024). The role of human-centered design in healthcare innovation: a digital health equity case study. J Gen Intern Med.

[20] Kalbach J (2020). Mapping Experiences: A Complete Guide to Creating Value through Journeys, Blueprints, and Diagrams.

[21] Gray D (2017). The Empathy Map Canvas.

[22] Pruitt J, Adlin T (2016). The Persona Lifecycle: Keeping People in Mind Throughout Product Design.

[23] Naranjo-Rojas A, Perula-de Torres LÁ, Cruz-Mosquera FE, Molina-Recio G (2023). Usability of a mobile application for the clinical follow-up of patients with chronic obstructive pulmonary disease and home oxygen therapy. Int J Med Inf.

[24] Davis FD (1989). Perceived usefulness, perceived ease of use, and user acceptance of information technology. MIS Q.

[25] Venkatesh V, Davis FD (2000). A theoretical extension of the technology acceptance model: four longitudinal field studies. Manage Sci.

[26] Fonteyn ME, Kuipers B, Grobe SJ (1993). A description of think aloud method and protocol analysis. Qual Health Res.

[27] (2018). Organic Law 3/2018, of 5 December, on Personal Data Protection and guarantee of digital rights. Official State Gazette.

[28] World Medical Association (2013). World Medical Association Declaration of Helsinki: ethical principles for medical research involving human subjects. JAMA.

[29] (1997). Convention for the protection of human rights and dignity of the human being with regard to the application of biology and medicine (convention on human rights and biomedicine). https://rm.coe.int/168007cf98.

[30] (1997). Universal declaration on the human genome and human rights. https://unesdoc.unesco.org/ark:/48223/pf0000110220.page=47.

[31] SMARTCLOTH - Odyssey.

[32] Andersen NS, Haugaard LH, Pedersen SB, Pedersen MS, Bygholm A (2020). Digital support for self-management in children with diabetes: understanding their needs and developing a design concept. Stud Health Technol Inf.

[33] Boggiss A, Consedine N, Hopkins S (2023). Improving the well-being of adolescents with type 1 diabetes during the COVID-19 pandemic: qualitative study exploring acceptability and clinical usability of a self-compassion chatbot. JMIR Diabetes.

[34] Hinshaw L, Basu A (2015). Technology use for problem solving in adolescent type 1 diabetes. Diabetes Technol Ther.

[35] Rewolinski JA, Kelemen A, Liang Y (2020). Type I diabetes self-management with game-based interventions for pediatric and adolescent patients. Comput Inf Nurs.

[36] Hadjiconstantinou M, Schreder S, Brough C (2020). Using intervention mapping to develop a digital self-management program for people with type 2 diabetes: tutorial on MyDESMOND. J Med Internet Res.

[37] Kaushal T, Katz LE, Marowitz ME, Joseph J, Ritter DP, Lipman TH (2019). 693-P: baseline characteristics and self-care behavior goals of adolescents with type 1 diabetes enrolled in MyDiaText. Diabetes.

[38] Nkhoma DE, Soko CJ, Bowrin P (2021). Digital interventions self-management education for type 1 and 2 diabetes: a systematic review and meta-analysis. Comput Methods Programs Biomed.

[39] Geoffrey A, Department of Nursing Kampala International University Uganda (2024). The effectiveness of digital health interventions on diabetes self-management and quality of life in adolescents with type 1 diabetes: a comprehensive review. RIJBAS.

[40] Chao EC (2024). Zooming in, then out: why we must apply human-centered design to transform diabetes technology. J Diabetes Sci Technol.

[41] Watterson JL, August A, Fourt J, Brettler T (2022). The role of human-centered design in insulin pen innovation and the future of insulin delivery. J Diabetes Sci Technol.

[42] Hsieh MC, Kuo YM, Kuo YL (2023). Utilizing design thinking for effective multidisciplinary diabetes management. Healthcare (Basel).

[43] Bonet-Olivencia S, Carrillo-Leal J, Rao AH, Sasangohar F (2024). User-centered design of a diabetes self-management tool for underserved populations. J Diabetes Sci Technol.

[44] Holtz BE, Murray KM, Hershey DD (2017). Developing a patient-centered mHealth app: a tool for adolescents with type 1 diabetes and their parents. JMIR mHealth uHealth.

[45] Pike JM, Moore CM, Yazel LG (2021). Diabetes prevention in adolescents: co-design study using human-centered design methodologies. J Particip Med.

[46] Pulman A, Taylor J, Galvin K, Masding M (2013). Ideas and enhancements related to mobile applications to support type 1 diabetes. JMIR mHealth uHealth.

