# Supplementary material for: SMARTCLOTH-Odyssey for Nutrition Education in Type 1 Diabetes: Development and Acceptability Study of a Gamified Web-Based Platform for Diabetes Nurse Educators Using a Human-Centered Design Approach
Source: JMIR Diabetes. 2026 Sep 25;11:e79648. doi: 10.2196/79648 (PMC13614223; doi:10.2196/79648)
Supplement: Supplementary file 1 [file diabetes-v11-e79648-s001.doc]

**Table
S1.**Adaptation
of the TAM^a^ model for the
SMARTCLOTH-Odyssey web acceptability study.

|  | **I fully agree** | **Moderately agree** | **Moderately disagree** | **Strongly disagree** |
| --- | --- | --- | --- | --- |
| Perceived usefulness | | | | |
| Using the web would improve my performance in nutrition education in DM^b^ |  |  |  |  |
| Using the web would improve my effectiveness in nutrition education in DM |  |  |  |  |
| Perceived ease of use | | | | |
| I think using the web has been easy |  |  |  |  |
| I think my interaction with the web was clear and understandable |  |  |  |  |
| Interaction with the web does not require mental effort |  |  |  |  |
| Learning to use the web was easy for me |  |  |  |  |
| I have found it easy to make the web do what I want it to do |  |  |  |  |
| Attitude toward use | | | | |
| I think it is a good idea to use a system like this to support me and reinforce diabetes education |  |  |  |  |
| I like the idea of using a web-based system for these educational activities |  |  |  |  |
| Using a web-based system in the practice would be a pleasant experience |  |  |  |  |
| Intention to use | | | | |
| If I had continuous access to the web, I intend to use it |  |  |  |  |
| Since I have access to the website, I want to use it as much as possible |  |  |  |  |

**Notes:** ^a^TAM: Technology Acceptance Model.^b^DM: diabetes mellitus.
